# Functional characterization of LotP from *Liberibacter asiaticus*

**DOI:** 10.1111/1751-7915.12706

**Published:** 2017-04-05

**Authors:** Flavia Loto, Janelle F. Coyle, Kaylie A. Padgett, Fernando A. Pagliai, Christopher L. Gardner, Graciela L. Lorca, Claudio F. Gonzalez

**Affiliations:** ^1^ Department of Microbiology and Cell ScienceGenetics InstituteInstitute of Food and Agricultural SciencesUniversity of Florida2033 Mowry roadPO Box 103610GainesvilleFL 32610‐3610USA; ^2^ PROIMI Planta Piloto de Procesos Industriales MicrobiológicosCONICETTucumánArgentina; ^3^ Department of Microbiology and Cell ScienceUndergraduate Research ProgramInstitute of Food and Agricultural SciencesUniversity of FloridaGainesvilleFLUSA

## Abstract

*Liberibacter asiaticus* is an unculturable parasitic bacterium of the alphaproteobacteria group hosted by both citrus plants and a psyllid insect vector (*Diaphorina citri*). In the citrus tree, the bacteria thrive only inside the phloem, causing a systemically incurable and deadly plant disease named citrus greening or Huanglongbing. Currently, all commercial citrus cultivars in production are susceptible to *L. asiaticus*, representing a serious threat to the citrus industry worldwide. The technical inability to isolate and culture *L. asiaticus* has hindered progress in understanding the biology of this bacterium directly. Consequently, a deep understanding of the biological pathways involved in the regulation of host–pathogen interactions becomes critical to rationally design future and necessary strategies of control. In this work, we used surrogate strains to evaluate the biochemical characteristics and biological significance of CLIBASIA_03135. This gene, highly induced during early stages of plant infection, encodes a 23 kDa protein and was renamed in this work as LotP. This protein belongs to an uncharacterized family of proteins with an overall structure resembling the LON protease N‐terminus. Co‐immunoprecipitation assays allowed us to identify the *Liberibacter* chaperonin GroEL as the main LotP‐interacting protein. The specific interaction between LotP and GroEL was reconstructed and confirmed using a two‐hybrid system in *Escherichia coli*. Furthermore, it was demonstrated that LotP has a native molecular weight of 44 kDa, corresponding to a dimer in solution with ATPase activity *in vitro*. In *Liberibacter crescens*, LotP is strongly induced in response to conditions with high osmolarity but repressed at high temperatures. Electrophoretic mobility shift assay (EMSA) results suggest that LotP is a member of the LdtR regulon and could play an important role in tolerance to osmotic stress.

## Introduction

Free‐living unicellular organisms are constantly exposed to life‐threatening challenges as a direct consequence of sudden, frequent and drastic changes in their environment. This fundamental principle is also true for bacterial species growing in close association with other organisms, which may provide shelter, nutrients or a suitable attachment substrate. Parasitic, commensal or mutualistic bacteria use similar mechanisms to survive the conditions encountered in the host environment. Insect‐vectored, phytopathogenic bacteria use different strategies to adapt their physiology to two organisms with remarkable physiological differences. The fundamental changes can be evaluated by quantifying fluctuations in gene expression and protein synthesis/modification, or by detecting variations in the amount/quality of the synthesized metabolites among others (Okinaka *et al*., 2002; Bouchart *et al*., 2007; Oshima *et al*., 2011; Yan *et al*., 2013).

*Liberibacter asiaticus* (from the *Rhizobiaceae* family) is an unculturable parasitic bacterium hosted by both citrus plants (and a few other species) and a psyllid insect vector (*Diaphorina citri*). In this work, we used the taxonomic nomenclature *L. asiaticus* observing the guidelines previously established for the use of the category Candidatus (Stackebrandt *et al*., 2002). Within the infected plant, this organism is restricted to the phloem, whereas within the insect, *L. asiaticus* can prosper in several tissues (Ammar *et al*., 2011). The phloem‐feeding insect works as a propagation vector, spreading the bacteria from infected trees to uninfected ones (Bové, 2006). Once the plant is infected, the bacteria thrive only inside the phloem, causing a systemically incurable and deadly plant disease named citrus greening or Huanglongbing. *L. asiaticus* infection drastically reduces citrus fruit production and quality, ultimately resulting in plant death in a few years (5–7 years) post‐infection. Currently, all commercial citrus cultivars in production are susceptible to *L. asiaticus*, representing a serious threat to the citrus industry worldwide (Wang and Trivedi, 2013).

A subset of characteristics distinctive of this pathosystem have made citrus greening a highly complex disease that is very difficult to successfully treat, including a large delay period (6 months to 3 years) from psyllid inoculation to manifestation of the first symptoms of infection in the plant; highly variable, patchy distribution of the bacteria in the roots and canopy (Bové, 2006; Ding *et al*., 2015; Louzada *et al*., 2016). These characteristics magnified by the technical inability to isolate and culture *L. asiaticus* have hindered progress in understanding the biology of this bacterium. Given this complex scenario, an ideal approach to develop a treatment for citrus greening is to impede the biological mechanisms that aid the bacteria in physiologically adapting to the plant host. Consequently, a deep understanding of the biological pathways involved in the regulation of host–pathogen interactions becomes critical to rationally design future strategies of control.

An important step towards understanding the genetic mechanisms essential to the *L. asiaticus*–host interaction was published a few years ago (Yan *et al*., 2013). Interestingly, when the abundance of *L. asiaticus* mRNA retrieved from infected trees was compared with mRNA obtained from the psyllid, 198 genes showed significant changes in expression. More than 90% of the *L. asiaticus* genes differentially expressed identified by Yan and coworkers were upregulated *in planta*. The three genes displaying the largest induction (> sevenfold), unfortunately, encoded for hypothetical or uncharacterized proteins, hindering the discernment of their true biological value. Two of those genes were previously associated with transport systems, whereas CLIBASIA_03135 was a conserved hypothetical protein that has not yet been characterized or associated with any known pathway.

To evaluate the biochemical characteristics and biological significance of CLIBASIA_03135, we have cloned the gene and purified the encoded protein (from now on named LotP). Co‐immunoprecipitation assays using His‐tagged LotP as a bait allowed us to identify the *Liberibacter* chaperonin GroEL as the main LotP‐interacting protein. The specific interaction between LotP and GroEL was reconstructed and confirmed using a two‐hybrid system in *Escherichia coli*. Furthermore, it was demonstrated that LotP has a native molecular weight of 44 kDa, corresponding to a dimer in solution with ATPase activity *in vitro*.

## Results

### *In silico* analysis and description

**Figure 1:**
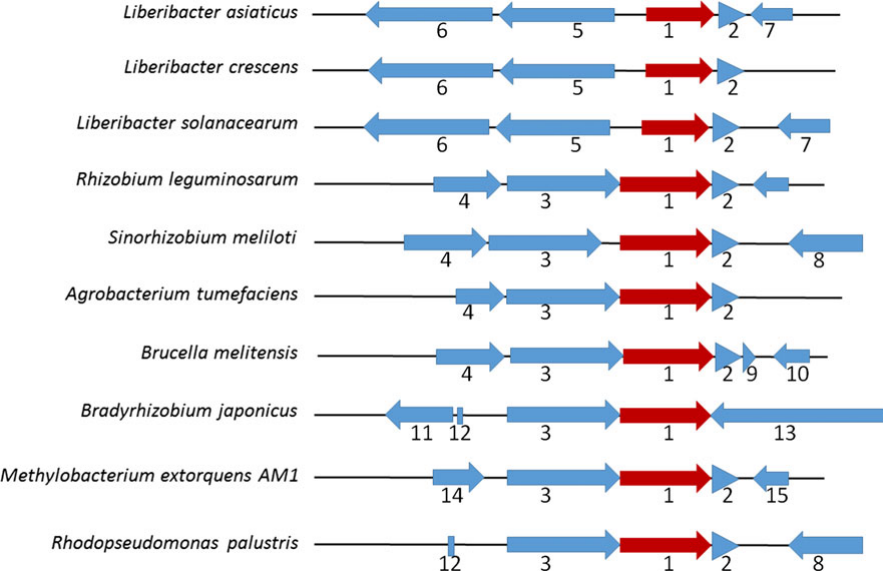
*CLIBASIA_03135* locus in *L. asiaticus* genome. The cluster is highly conserved in *Liberibacter* species as well as in other *Rhizobiaceae* members. (1) *CLIBASIA_03135* and homologues (red). (2) Hypothetical protein, acid/peroxide resistance. (3) Thioredoxin domain‐containing protein. (4) Aminoacyl‐tRNA editing enzyme. (5) Carboxynorspermidine decarboxylase (EC 4.1.1.96). (6) Carboxynorspermidine synthase (EC 1.5.1.43). (7) Hypothetical protein. (8) Ubiquinone biosynthesis hydroxylase, UbiH/UbiF/VisC/COQ6. (9) Hypothetical protein. (10) 3‐Isopropylmalate dehydrogenase (EC 1.1.1.85). (11) Putative oxidoreductase. (12) tRNA‐Gly. (13) Hypothetical protein. (14) Hypothetical protein. (15) Hypothetical protein.

The LotP‐encoding gene, *CLIBASIA_03135*, is located in a highly conserved cluster in the genome of all sequenced species of the *Liberibacter* genus. This gene is also conserved in members of the *Rhizobiaceae* family. In *Liberibacter* genomes, *CLIBASIA_03135* is associated with a small gene, 192 base pairs (bp) long, located directly downstream and separated by a region of only 14 bp (Fig. 1).

**Figure 2:**
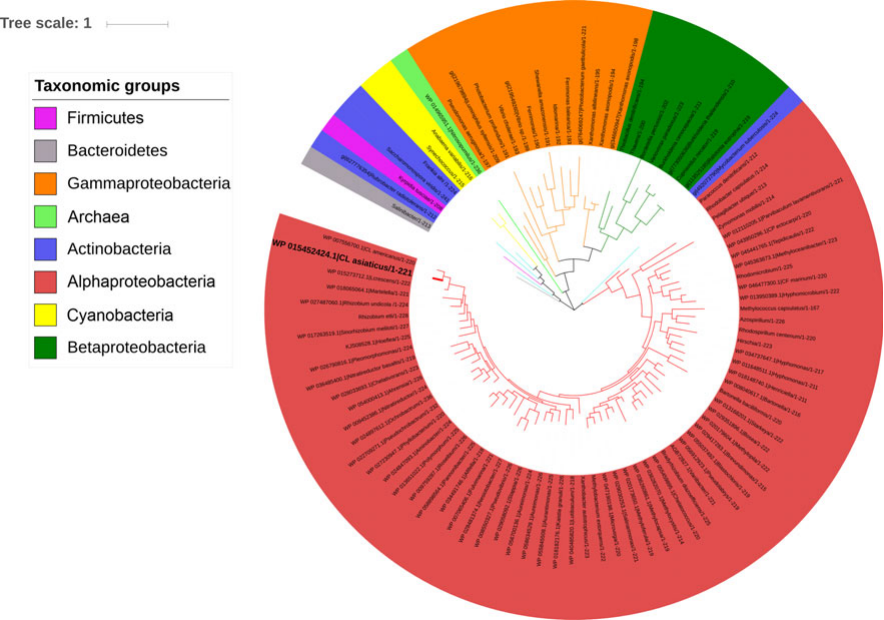
Phylogenetic analysis of LotP. The evolutionary history of LotP was inferred by using the maximum‐likelihood method based on the JTT matrix‐based model. The phylogenetic tree with the highest log likelihood (−15397.4801) is shown. Initial trees for the heuristic search were obtained automatically by applying neighbor‐joining and BioNJ algorithms to a matrix of pairwise distances estimated using a JTT model. The topology with superior log‐likelihood value was selected, the branch lengths measured in the number of substitutions per site. The analysis involved 96 amino acid sequences. All positions containing gaps and missing data were eliminated. Evolutionary analyses were conducted in MEGA7.

*CLIBASIA_03135* encodes for a hypothetical, uncharacterized, 221‐amino‐acid protein. A BLAST analysis using LotP's linear amino acid sequence as a query indicated a high degree of conservation in homologous proteins encoded in the *Rhizobiaceae* family, including *Liberibacter solanacearum* (86% identity), *Liberibacter africanus* (85% identity), *Liberibacter americanus* (75% identity), *Liberibacter crescens* (64% identity), *Sinorhizobium meliloti* and *Agrobacterium tumefaciens* (56% identity in both cases). LotP homologues are also present in other taxa like *Burkholderia*,* Mycobacterium* and *Pseudomonas*. LotP's evolutionary history was inferred by using the maximum‐likelihood method based on a JTT matrix‐based model. Our analysis indicated a vast distribution of LotP in the alphaproteobacteria group with scattered presence in members of gammaproteobacteria and betaproteobacteria. Interestingly, 35% of the microorganisms identified in the analysis were plant‐associated bacteria and half of those species are well‐known plant pathogens (Fig. 2).

**Figure 3:**
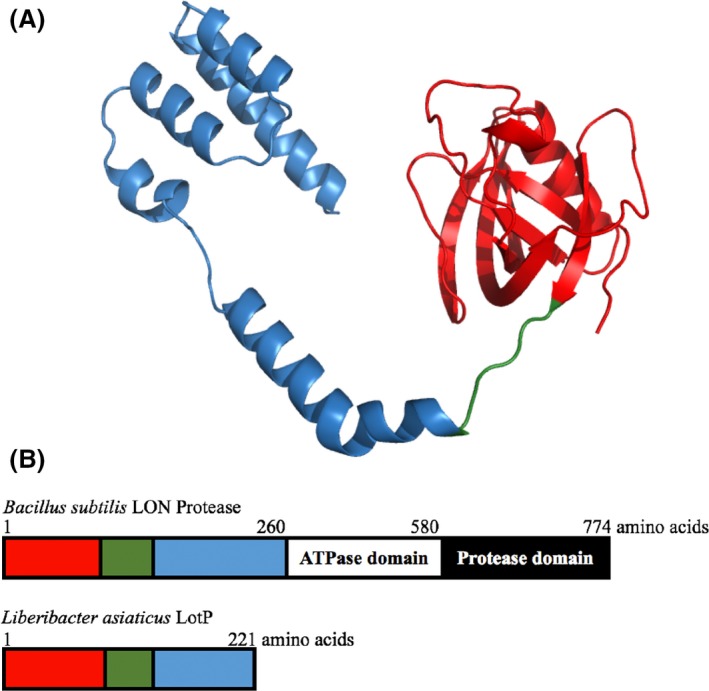
*In silico* structural analysis of LotP. A. Ribbon representation of predicted LotP model. The model was constructed based on the structural similarities with *B. subtilis* N‐terminus LON protease (PDB: 3M65). The globular domain is depicted in red; a flexible loop in green connects this domain with the four‐helix bundle coloured in blue. B. Schematic representation of the domains present in LotP and in *B. subtilis* LON protease. LotP conserved scaffolds are represented with the same colours used in the predicted model described above. *B. subtilis* LON protease N‐terminal domain is 40 residues longer, the primary sequence not being conserved. LON protease is composed of two additional domains, the ATPase and protease in the C‐terminus of the protein.

A structural‐based search, using Phyre2 and SWISS‐MODEL servers, identified the N‐terminal domain of *Bacillus subtilis* ATP‐dependent protease La1 (LON Protease) as the protein with the highest structural similarities. A predicted LotP model was obtained using this La1 domain (PDB: 3M65) that comprises the first 209 amino acids of the LON protein. This peptide possesses minimal sequence identity with LotP (identity = 18%) but a comparable tertiary structure. The structural model constructed, with 90% of sequence coverage, consists of two subdomains: one globular domain formed by the first 120 amino acids of the N‐terminal region and a distant α‐helical domain shaped by a four‐helix bundle (Fig. 3). These two regions are connected by a flexible loop formed by six amino acids. Consequently, and based only on structural similarities with this small domain, the product of the *CLIBASIA_03135* gene was previously annotated in protein databases as an LA1 aminopeptidase, also known as a LON protease. However, the total length of the amino acid sequence of the LON protease is four times longer than LotP and two critical LON protease domains are absent in LotP, the central ATPase motif (200–240 amino acids) and the C‐terminal protease domain (240–300 amino acids). Collectively, the *in silico* evidence retrieved suggests that LotP belongs to a different, as of yet uncharacterized protein family.

### Identification of LotP protein interaction partners

The LON protease is a key component of the cellular protein control system (Bezawork‐Geleta *et al*., 2015; Cho *et al*., 2015). This system is responsible for maintaining cell protein quality (proper folding maintenance and optimal biological function). The N‐terminal module of the LON protease is involved in recognition and binding of the substrates to be hydrolysed (Roudiak and Shrader, 1998; Chir *et al*., 2009; Li *et al*., 2010; Wohlever *et al*., 2014). Consequently, because of the high similarities in protein folding, we hypothesize that the biological role of LotP may depend upon interaction with other intracellular proteins. With the aim of testing our hypothesis, *CLIBASIA_03135* was cloned, His‐tagged, and the recombinant His_6X_‐LotP protein was purified by affinity chromatography as previously described (Pagliai *et al*., 2014). The purified protein was dialysed in the presence of MgCl_2_, which was critical to maintain the protein in solution (see Experimental Procedures section).

**Figure 4:**
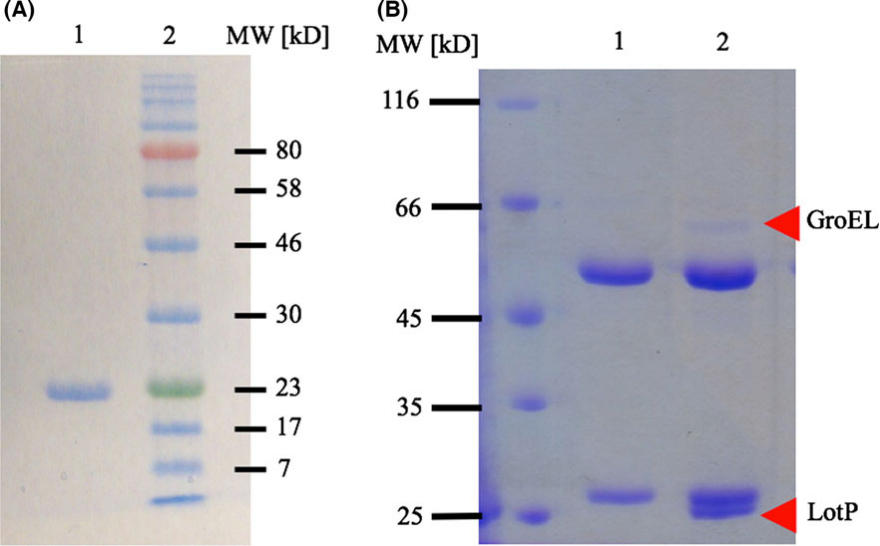
Identification of LotP interaction partners. A. Lane 1, SDS‐PAGE, His_6X_‐LotP purified by affinity chromatography. Lane 2, Color Plus pre‐stained MWM (New England BioLabs). B. SDS‐PAGE showing the results of the immunoprecipitation assay Lane 1: control, *L. crescens* cell‐free extract was mixed with monoclonal anti‐His_6X_ antibody in the absence of LotP. The light and heavy chains of the monoclonal antibody are visible. Lane 2: the immunoprecipitation was carried out with the same monoclonal antibody using His_6X_‐LotP as a bait and *L. crescens* cell‐free extract. LotP ~23‐kDa band and *L. crescens* GroEL (~60 kDa) are indicated with arrows. In this gel, EZ‐run was used as molecular weight marker (Fisher Scientific).

The purified protein showed an apparent molecular weight (MW) of 23 kDa in denaturing SDS‐PAGE (Fig. 4A). The native molecular mass was 44 kDa as determined by size‐exclusion chromatography using a Superose 12 resin, while similar results were obtained in the presence of ATP (Fig. S3). These results indicate that LotP is a dimer in solution and, in contrast to LON protease, its dimerization is not affected by ATP.

Cell‐free extract of *L. crescens*, the closest culturable relative to *L. asiaticus*, was used as the sample to perform immunoprecipitation assays. Pure His_6X_‐LotP was used as a bait and the interaction complex was recovered using magnetic beads coated with anti‐His‐tag antibodies. The complexes obtained were analysed via SDS‐PAGE (Fig. 4B). Several minor protein bands (~5) were observed as a result of the co‐immunoprecipitation but only one was prominent, with an apparent monomeric MW of 60 kDa. The samples were analysed by mass spectrometry, and the results obtained are summarized in Table 1. Collectively, the results suggest that LotP interacts with proteins involved in the prokaryotic cellular stress response, with the chaperone GroEL being a prominent target. Equivalent assays were carried out using *S. meliloti* (Fig. S2) and *E. coli* cell‐free extracts and comparable results were obtained.

**Table 1:** LotP Immunoprecipitation assays: protein identified with MS/MS

Protein identified	Accession Number (gi)	Molecular weight	Total unique peptide count
Hsp40 chaperone	940652580	41 kDa	5
Serine endoprotease	940652534	49 kDa	2
ClpX	254039948	47 kDa	5
GroEL	254040526	58 kDa	34

Furthermore, the protein–protein interaction between LotP and GroEL was assessed by cloning the genes encoding the interacting proteins in a two‐hybrid system (Vallet‐Gely *et al*., 2005). The assay was carried out using *E. coli* as previously described (Wrench *et al*., 2013). The gene encoding LotP was fused to the Zif protein and the *CLIBASIA_03720* gene (encoding GroEL) was fused to the ω subunit of the RNAP; the reciprocal fusions were also constructed and included in the assay (see Table 2 for strain descriptions). In this system, the target proteins fused to the Zif protein and ω subunit of the RNAP must physically interact in order to stimulate transcription of the *lacZ* gene, encoding the β‐galactosidase, in the *E. coli* reporter strain. This physical interaction is then quantitatively measured by monitoring the resulting amount of β‐galactosidase activity produced in each strain. Physical interaction between the target proteins results in increased β‐galactosidase activity compared with control strains. The strains were grown in MOPS minimal media, the chimerical proteins induced with IPTG, and the β‐galactosidase activity was determined. The strains FL03 and FL05 carrying both recombinant plasmids demonstrated a significantly higher β‐galactosidase (1988 ± 49 and 1249 ± 16 AU, respectively) activity compared with strains carrying empty plasmids (941 AU). The protein–protein interaction was detected in a range of temperature (30–42°C). The values described in Table 3 belong to the enzymatic activities obtained at 37°C. The β‐galactosidase activity at 25°C was significantly lower (170 AU), probably due to the slow growth of *E. coli*. These results, the co‐immunoprecipitation and higher β‐galactosidase activity, are in support of the physical interaction between these two *L. asiaticus* proteins. The results are summarized in Table 3.

**Table 2:** Strains and plasmids used in this study

Strains	Relevant genotype	References
*E. coli* DH5α	ϕ80 d*lac*ZΔM15Δ(*lac*ZYA‐*arg*F)U169 *rec*A1 endA1 hsdR17 (rK− mK+) supE44 thi‐1*gyr*A *rel*A1	Laboratory stock
*E. coli* BL21 (DE3)	F− *omp*T *gal dcm lon hsd*SB(r_B−_ m_B−_) λ(DE3 [*lac*I *lac*UV5‐T7 *gene* 1 *ind*1 *sam*7 *nin*5])	Stratagene
*E. coli* JM109	*end*A1, *rec*A1, *gyr*A96, *thi*‐1, *hsd*R17 (r_K−_ m_K+_), relA1, supE44, Δ(lac‐*pro*AB), [F’ traD36,proAB, laqIqZΔM15]	New England Biolabs
*E. coli* KDZif1ΔZ	*ara*D (*gpt*‐*lac*)5, *rps*L (Str^r^), Δ*spo*S3::*cat* (Cam^r^) [F’ *lac*I^q^ (Z321(‐61) lacZYA*) Kan^r^]	(Vallet‐Gely *et al*., 2005)
*E. coli* BW25113	F^−^, DE(*ara*D‐*ara*B)567, *lac*Z4787(del)::rrnB‐3, LAM^−^, rph‐1, DE(rhaD‐rhaB) 568, hsdR514	Keio collection
*E. coli* JW4103	F^−^, *Δ(araD‐araB)567*,* ΔlacZ4787*(::rrnB‐3), *λ* ^*−*^, *rph‐1*,* Δ(rhaD‐rhaB)568*,* ΔgroL768::kan*,* hsdR514*	Keio collection
*L. crescens* BT‐1	Standard strain (Wild Type).	(Leonard *et al*., 2012)
*S. meliloti* 1021	*exp*R102::IS*Rm*2011‐1*exp*R.Sm^r^	(Galibert *et al*., 2001)
FL01	KDZif1ΔZ pACTR‐Zif, pBRGP‐ω. Tetr, Amp^r^. Kan^r^	This work
FL02	KDZif1ΔZ pACTR‐*CLIBASIA_03135*‐Zif, pBRGP‐ω. Tetr, Amp^r^. Kan^r^	This work
FL03	KDZif1ΔZ pACTR‐Zif, pBRGP‐*CLIBASIA_03135*‐ω. Tetr, Amp^r^. Kan^r^	This work
FL04	KDZif1ΔZ pACTR‐*CLIBASIA_03720*‐Zif, pBRGP‐*CLIBASIA_03135*‐ω. Tetr, Amp^r^. Kan^r^	This work
FL05	KDZif1ΔZ pACTR‐*CLIBASIA_03135*‐Zif, pBRGP‐*CLIBASIA_03720*‐ω. Tetr, Amp^r^. Kan^r^	This work
FL06	KDZif1ΔZ pACTR‐*CLIBASIA_03720*‐Zif, pBRGP‐ω. Tetr, Amp^r^. Kan^r^	This work
FL07	KDZif1ΔZ pACTR‐Zif, pBRGP‐*CLIBASIA_03720*‐ω. Tetr, Amp^r^. Kan^r^	This work
Plasmids	Characteristics	Reference
p15TV‐L	Expression vector Amp^r^.	(Pagliai *et al*., 2010)
pBRGP‐ω	Translational fusion vector	(Vallet‐Gely *et al*., 2005)
pACTR‐AP‐Zif	Translational fusion vector. Tet^r^	(Vallet‐Gely *et al*., 2005)
pBAD24	Expression vector, arabinose inducible promoter, Amp^r^	(Guzman *et al*., 1995)
pFL02	pACTR‐*CLIBASIA_03135*‐Zif. Tet^r^	This work
pFL03	pBRGP‐*CLIBASIA_03135*‐ω. Amp^r^	This work
pFL04	pBRGP‐*CLIBASIA_03720*‐ω. Amp^r^	This work
pFL05	pACTR‐*CLIBASIA_03720*‐Zif. Tet^r^	This work
pFL06	pBAD24‐*CLIBASIA_03135*. Amp^r^	This work

**Table 3:** Analysis of LotP interactions with GroEL

Strains	Plasmids and fused proteins		β‐Galactosidase activity
	pBRPG‐ω	pACTR‐AP‐Zif	
FL01	–	–	941 ± 44
FL02	–	LotP	811 ± 12
FL03	LotP	GroEL	1988 ± 49*
FL04	–	GroEL	866 ± 57
FL05	GroEL	LotP	1249 ± 16*
FL06	GroEL	–	1030 ± 17

LotP = *CLIBASIA_03135* gene; GroEL = *CLIBASIA_03720* gene. The mean ± SD is shown. ANOVA test indicated statistically significant variation in mean β‐galactosidase activity across strains (*F*_5,18_ = 232.7; *P*‐value = 1.07e‐15). Strains followed by an asterisk (*) are significantly different from all other strains according to Tukey's HSD test (α = 0.05). The experiments were performed in triplicate.

### LotP displays phosphatase activity in solution

**Figure 5:**
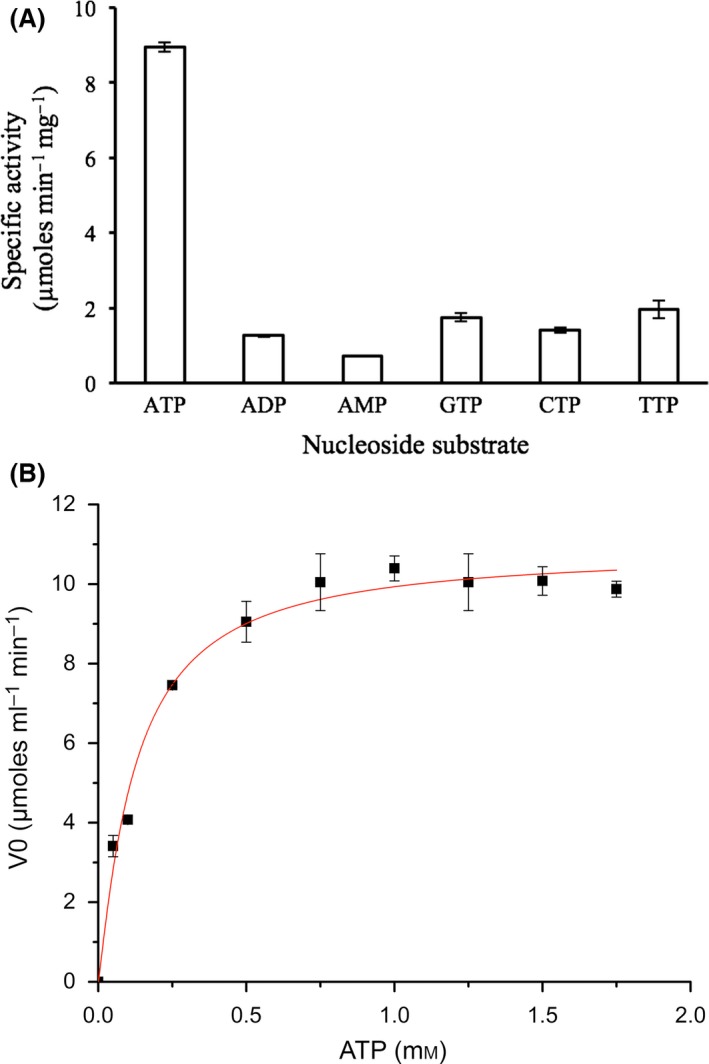
LotP phosphatase activity determinations. A. Phosphatase activity profile using several nucleosides as enzyme substrate. Typical enzyme reaction contains 1 mM of each substrate and 2–10 μg ml^−1^ of enzyme. B. Steady‐state kinetic characterization of ATPase activity. Initial rates of the reactions were determined at fixed concentrations of ATP, and the P*i* released was quantified by using Malachite green reagent as previously described (Crowe *et al*., 2010).

*In silico* analyses were not conclusive in predicting any catalytic activity associated with this protein. Laboratory assays were performed to evaluate the two activities classically associated with the LON protease, namely protease and phosphatase activity. No proteolytic activity was detected, but LotP showed a weak activity towards *p*‐NPP (phosphatase model substrate) (Proudfoot *et al*., 2008). In a subsequent enzymatic assay, LotP's activity towards several nucleosides was tested in parallel. Malachite green was used to detect the P_*i*_ released by the enzyme action (Crowe *et al*., 2010). The results demonstrated that LotP preferentially used ATP as an enzyme substrate (Fig. 5A). The ATPase activity was consistent in a wide range of pH (5.5–9), and the optimal temperature for *in vitro* catalysis was 50°C. The enzyme demonstrates classical hyperbolic saturation kinetics with an apparent *V*_max_ *= *10.2 μMoles min^−1^ and a *K*_*m*_ = 133.98 μM (Fig. 5B). LotP alone, or used in combination with purified GroEL, showed no chaperone activity *in vitro* (data not shown).

### LotP is able to interact with *E. coli* proteins affecting cell viability

**Figure 6:**
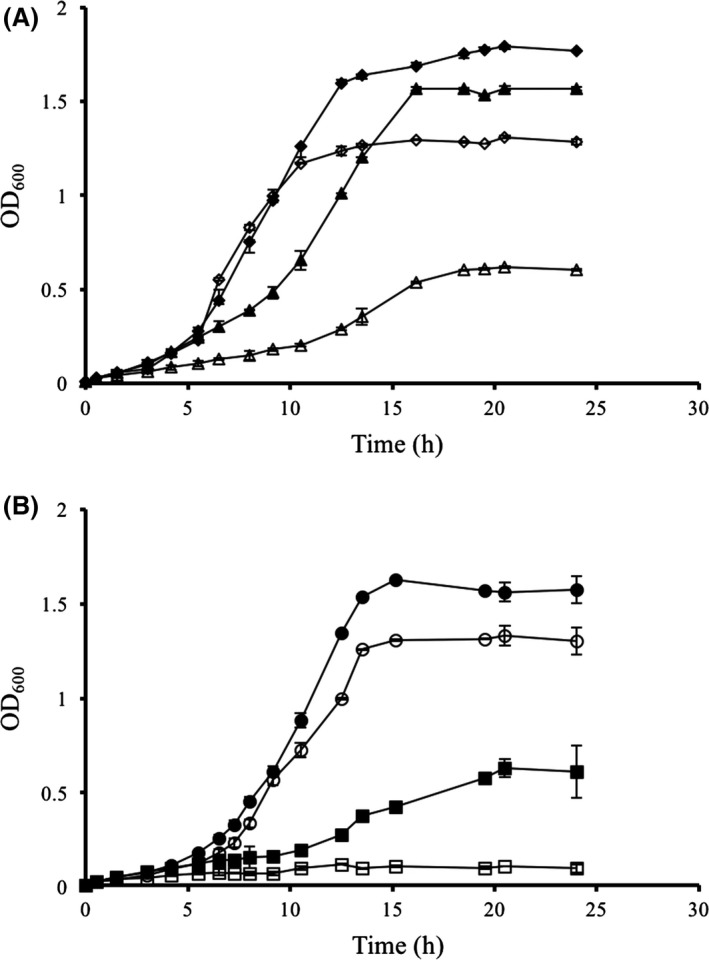
Effect of temperature on the growth of *E. coli* strains expressing LotP from an inducible expression vector. The gene *CLIBASIA_03135* was cloned in pBAD24. The cells were grown in MOPS minimal medium with glycerol as carbon source. LotP expression was induced with 10 mM l‐arabinose starting at the beginning of the assay. The assays were carried out at 37°C (closed symbol) and 42°C (open symbols). A. BW25113 (WT): pBAD empty plasmid (diamonds, ♦ – ♢); pBAD‐LotP (triangles, ▲ – ▵); B. JW4103 *(*Δ*groEL)*: pBAD empty plasmid (circles, ● – ○); pBAD‐LotP (squares, ■ – □). Plotted values represent the mean ± SD of three biological replicates.

The main biological role of bacterial GroEL is related to the maintenance of correct protein folding during stress (Georgopoulos, 2006; Hayer‐Hartl *et al*., 2016). Specifically, this protein protects cell integrity by refolding denatured proteins, mostly those proteins affected by heat denaturation (Cho *et al*., 2015). With the aim of assessing the biological effects of LotP expression on cell growth during temperature stress, LotP was cloned in the pBAD24 expression vector (Guzman *et al*., 1995). The protein was expressed in the *E. coli* wild‐type strain, BW25113, and the isogenic JW4103, a mutant with impaired ability to respond to heat shock due to lower levels of GroEL production. The growth of the wild‐type strain expressing LotP at 42°C was less than the growth observed at 37°C. In both conditions, the strains expressing LotP grew slower since the beginning of the assay (Fig. 6A). This kind of effect is typical of strains with flawed adaptation to important environmental challenges, such as thermal stress in this case. The effect of temperature was drastic when LotP was expressed in the mutant strain. *E. coli* JW4103 expressing LotP was unable to grow at 42°C after 24 h of incubation (Fig. 6B). To explain these results, a new immunoprecipitation assay was performed, this time using *E. coli* cell‐free extract obtained from both strains (BW25113 and JW4103). Mass spectrometry analysis of the proteins recovered demonstrates that LotP is also able to interact with *E. coli* GroEL. Surprisingly, seven other proteins, including DnaJ and ClpX, co‐precipitate with LotP when the JW4103 cell‐free extract was used. Therefore, we hypothesize that LotP interacts with GroEL in *E. coli*. However, in the absence of GroEL, other proteins essential for survival during temperature challenges can interact with LotP, affecting the overall cell viability (Fig. S1).

### LotP transcription is repressed at high temperatures in *L. crescens*

**Figure 7:**
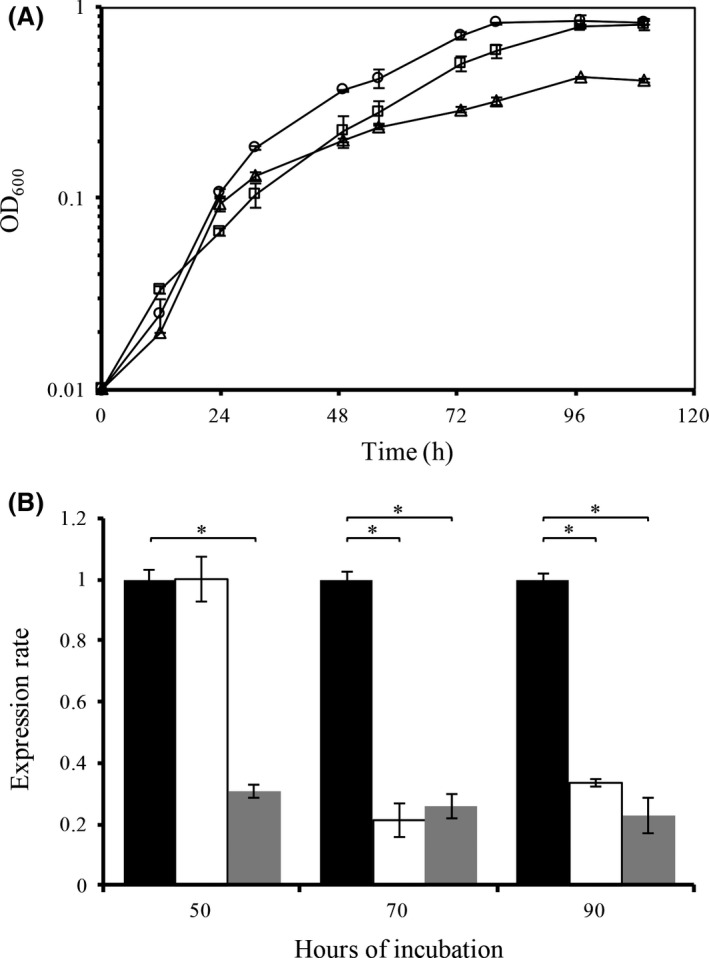
Effect of temperature on mRNA levels of LotP. A. *L. crescens* growth curve: 25°C (circles, ○), 30°C (squares, □) and 32°C (triangles, ▵). Plotted values represent the mean ± SD of three biological replicates. B. The mRNA transcripts of *B488_12950* (encoding a LotP homologue) were quantified by qRT‐PCR. The expression rate was normalized using the expression of the *L. crescens* 16S rRNA as internal standard using the primers depicted in Table 4. Samples were obtained at 50, 70 and 90 h of incubation. The temperature of incubation for each case was 25°C (black bars), 30°C (white bars) and 32°C (grey bars). Expression rate values represent the mean normalized fold change ± SD as compared with the control, performed in quadruplicates. Statistical significance was determined using a Student's *t*‐test (*P < 0.05). All the assays were conducted in a rotary shaker at 200 rpm.

*Liberibacter asiaticus* cannot be cultured in a laboratory setting, but the closely related *L. crescens* can be used as surrogate strain (Pagliai *et al*., 2014; Lai *et al*., 2016). In general, *Liberibacter* species are temperature sensitive, with an optimal growth temperature of 25°C (Fagen *et al*., 2014). Consequently, heat stress was induced by culturing the bacteria at 30 and 32°C (Fig. 7A). The highest temperature evaluated (32°C) was the maximal temperature tolerated by *L. crescens* in the culture conditions used. A comparable growth kinetic was obtained for all temperatures of incubation assayed. The rate of transcription for *lotP* was drastically affected by temperature; fivefold repression was observed when the temperature was higher than 30°C (Fig. 7B). The results obtained in *L. crescens* are in agreement with the results described in *E. coli*. Altogether, the results suggest that the biological role of LotP is not directly related to the resistance of this bacterium to high temperatures.

### LotP is highly induced by conditions with high osmolarity in *L. crescens*

**Figure 8:**
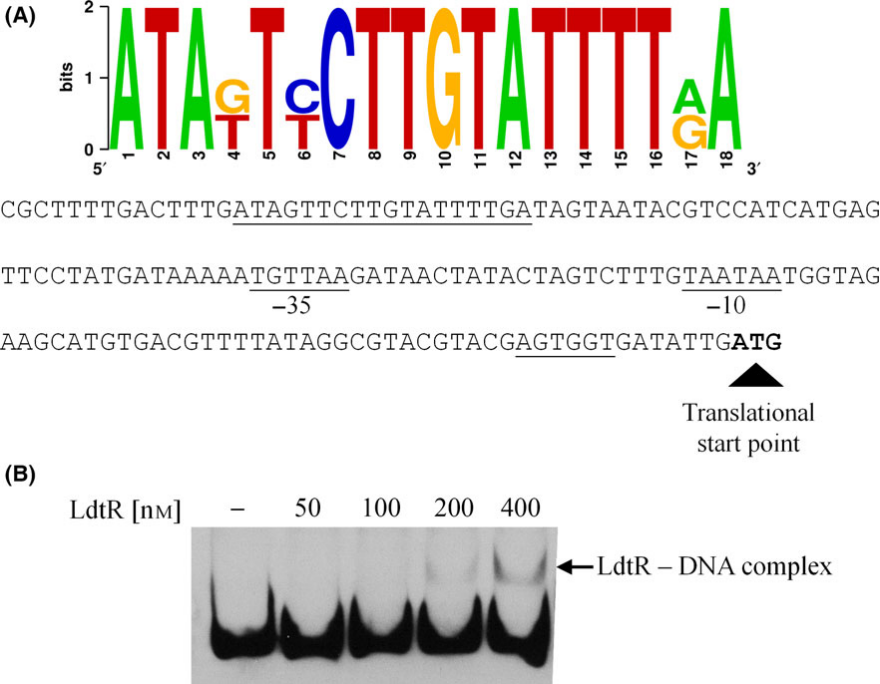
LdtR binds in the LotP promoter A. The LdtR binding sequence was estimated by RegPredict software using a known position weight matrix based on data previously published (Pagliai *et al*., 2014). B. EMSAs were conducted with increasing concentrations of LdtR as shown in the top of each figure. The arrow denotes the shift obtained using 200 and 400 nanomolar of purified protein.

In search of potential regulatory sequences controlling LotP expression, a direct analysis of the DNA region immediately upstream of the *lotP* gene was performed using the binding motif determined by Pagliai *et al*. (2014). A putative binding motif for LdtR, a recently characterized transcription factor, was found 117 base pairs upstream of the LotP translational start codon (Fig. 8A). LdtR is a MarR‐family transcriptional regulator governing the expression of several *L. asiaticus* genes. The LdtR regulon modulates the expression of a subset of proteins facilitating osmotic tolerance in *L. asiaticus*. The predicted DNA binding region was amplified, labelled with biotin and used in electrophoretic mobility shift assay (EMSA) as the LdtR target. The binding analysis showed that LdtR recognizes and binds the DNA sequence predicted via *in silico* analysis (Fig 8B). These results confirm that LotP is a member of the LdtR regulon.

**Figure 9:**
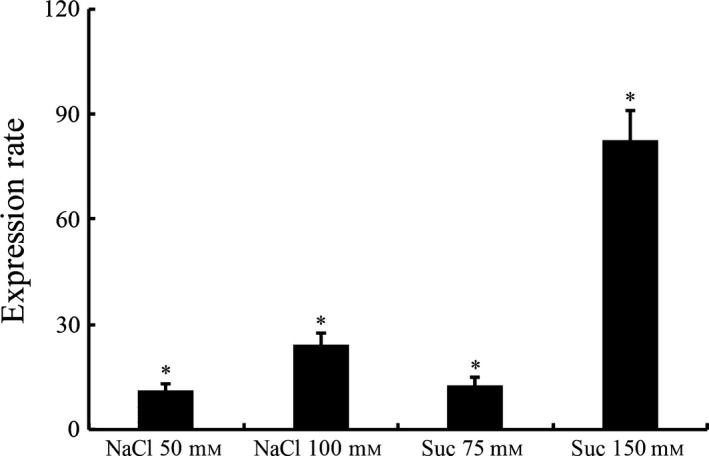
LotP is induced under high osmotic conditions. The expression level of LdtR was assessed in *L. crescens* growing on BM7 culture media amended with NaCl and sucrose at the indicated concentrations (*x*‐axis). The assays were performed in a rotary shaker at 200 rpm and 25°C. mRNA was extracted as described and the expression rate measured by qRT‐PCR. The 16S rRNA expression was used as internal standard. Expression rate values represent the mean normalized fold change ± SD as compared with the control, performed in quadruplicates. Statistical significance was determined using a Student's *t*‐test (*P < 0.05).

The apparent affinity obtained was directly comparable to the affinity originally described for this LdtR promoter. This result suggests that LotP expression could be induced in response to high concentrations of osmotically active solutes. To verify the biological significance suggested by this *in vitro* assay, LotP expression was quantified in samples obtained from *L. crescens* exposed to high concentrations of NaCl and sucrose. In this strain, the LotP homologue as well as its regulatory region is highly conserved. *L. crescens* can tolerate up to 150 mM NaCl and 200 mM of sucrose in the culture media; therefore, the concentrations used to increase the osmolarity did not significantly affect the growth of *L. crescens* as previously described (Pagliai *et al*., 2014). The cells were grown in BM7 culture media amended with NaCl (50 and 100 mM) or sucrose (50 and 100 mM). The results obtained indicate that the rate of expression of LotP increased up to 90 ± 10 times in the presence of 100 mM sucrose and 30 ± 3 times with the same concentration of NaCl (Fig. 9). These results suggest that LotP is a member of the LdtR regulon and could play an important role in tolerance to high osmotic pressure, an environmental condition encountered in the citrus phloem sap.

## Discussion

The analysis of LotP primary structure, linear sequence, indicates that it is highly conserved and well represented in bacterial species frequently associated with plants. Structural analysis of the protein indicates that it displayed remarkable structural homology with the amino‐terminal region of the LON protease (240 amino acids long) (Duman and Löwe, 2010; Li *et al*., 2010). Based on these structural similarities, LotP was systemically annotated as an ATP‐dependent LON protease in all sequenced genome databases; however, the LON protease is an ATP‐dependent aminopeptidase composed by six units of 90 kDa each (760–800 amino acids) (Lin *et al*., 2016).

These contrast with the characteristics and native molecular mass (44 kDa) determined for LotP in solution. Additionally, LotP is missing two critical LON modules: the ATP‐binding module with a characteristic Walker motif and the proteolytic module located in the carboxy‐terminal region. The phylogenetic relationships suggest that LotP evolution belongs to a different lineage of proteins unrelated to the LON protease. Altogether, these features suggest that LotP belongs to a different family of proteins with a protein fold similar to the amino‐terminal module of the LON protease.

The structural similarities described are likely the consequence of convergent evolution. The function of the LON protease's N‐terminal module is the identification of proteins (substrates) to be hydrolysed by the proteolytic domain. Based on the similarity between the folds of the N‐terminal module of LON protease and LotP, we expected that LotP might have a similar function. In absence of direct biological data to guide our research, the immunoprecipitation assay to identify LotP protein partners was the best starting strategy. The recovery of a 60 kDa protein in the immunoprecipitation assay, later identified as GroEL (34 peptides identified by GC/MS), suggests that LotP could have a central role in the *L. asiaticus*–*Citrus sinensis* pathosystem. GroEL belongs to the chaperonin protein family, CPN60, that is an evolutionarily conserved family associated with the maintenance and refolding of intracellular proteins (Mayhew *et al*., 1996; Guisbert *et al*., 2008; Hayer‐Hartl *et al*., 2016). GroEL is also indispensable for several nodulating species of *Rhizobiaceae* which encode up to seven copies per genome (Fischer *et al*., 1993; Lund, 2009). In other cases, it behaves as a potent MAMP (microbial‐associated molecular pattern), triggering plant resistance mechanisms (Chaudhary *et al*., 2014). It has been directly associated with the modulation of several microbial–host interactions and directly connected to a baffling variety of biological roles. One of the most intriguing examples involves *Buchnera aphidicola*, a gram‐negative bacterium of the gammaproteobacteria group. This bacteria is an aphid endosymbiont that depends upon the overexpression of GroEL for long‐term maintenance of its relationship with the insect host (Wilcox *et al*., 2003). However, when *B. aphidicola* purified GroEL is injected into vegetable tissues, it triggers the expression of the defensive arsenal of the plant. A complete overview of the biological significance of GroEL has been compiled in two excellent reviews (Henderson *et al*., 2013; Kupper *et al*., 2014). Understanding the fundamental role of GroEL in this system could shed light on the biological meaning of LotP. In *L. asiaticus,* the transcription of GroEL slightly increases when the bacteria is transferred to the plant (Yan *et al*., 2013). To minimize the plant response to *L. asiaticus*, GroEL should be silenced or ‘protected’ to evade the plant defence system. We hypothesize that LotP's biological role could be to minimize the plant's immune response, protecting *L. asiaticus* GroEL. In this way, the plant's resistance will be hampered, permitting infection by *L. asiaticus*. As of yet, this question remains to be properly answered.

Recombinant strains of *E. coli* expressing *L. asiaticus* LotP are more sensitive to high temperatures and the bacterial growth is directly affected. In the bacterial strains where the main target of LotP is lower (strain JW4103), LotP interacts with a subset of stress‐related proteins and impedes cellular growth at 42°C. The results observed in *L. crescens* correlate with those obtained in *E. coli. *The transcriptional repression of LotP in *L. crescens* incubated at elevated temperatures clearly indicates that LotP is not enhancing the protective role of GroEL when cells are exposed to high temperatures. Although *L. crescens* encodes a functional *rpoH* gene, the transcriptional rate of GroEL does not change or shows a negligible decrease when the strain is cultured at 32°C. This suggests, as was reported in many primary symbionts, that the main function of these proteins in *L. asiaticus* is not synergistic chaperone/co‐chaperone activity (Stoll *et al*., 2009). Indeed, *L. asiaticus* is especially sensitive to elevated temperatures and controlled heat treatment of citrus trees is the only method used to transiently decrease the bacterial titre in inflected plants (Hoffman *et al*., 2013). Collectively, these data suggest an overall post‐translational regulatory role for LotP through protein–protein interactions. In this context, the ATPase activity described herein became critical to provide the energy necessary to release the interacting proteins according to the needs of the cell (Fenton *et al*., 1994). Alternatively, LotP may help the bacteria to cope with the stress produced by heat. LotP transcription is repressed when the bacterium grows at high temperatures, which will result in lower LotP synthesis releasing active GroEL when necessary. This possibility is in agreement with the evidence herein displayed. However, this is difficult to evaluate due to the narrow range of growth temperature for *L. crescens* and the impossibility to culture *L. asiaticus* in a laboratory setting.

The gene encoding LotP is one of the few genes in *L. asiaticus* that is highly transcribed in mRNA samples obtained from infected citrus. The transcriptional rate increased nine times when compared with samples collected from infected psyllids (Yan *et al*., 2013). The *in silico* identification of highly conserved LdtR binding sequences along with the associated *in vitro* DNA binding assays suggests a direct regulation of LotP by this recently characterized transcription factor (Pagliai *et al*., 2014, 2015). The exceptionally high expression (up to 90 times) obtained when *L. crescens* is cultured under hyperosmotic conditions confirmed that LotP belongs to the LdtR regulon. It may, directly or indirectly, help the cells to tolerate the high osmotic pressure of the phloem sap. The inability to culture *L. asiaticus* hampers the possibility of directly addressing this question by using mutant strains. The biological role of GroEL on high saline and high osmotic conditions varies depending on the bacterial species analysed (Susin *et al*., 2006; Gunasekera *et al*., 2008; Rao *et al*., 2013). Overall, the role of GroEL cannot be directly linked to protein refolding as it can with cells growing at high temperature.

We hypothesize that the biological significance of LotP is directly related to the moonlighting activity of GroEL. The GroEL protein is extremely important in almost all insect‐vectored pathosystems studied and *L. asiaticus* should not be an exception. Several questions need to be properly addressed before completely understanding the true biological value of this protein–protein interaction. However, the results described herein represent one step towards improved comprehension of the complex molecular events driving the relationship between *L. asiaticus* and the host.

## Experimental procedures

### *In silico* analysis

Homologues of CLIBASIA_03135 were obtained using two iterations of a PSI‐BLAST search against a non‐redundant sequence database at the NCBI (www.blast.ncbi.nlm.nih.gov/). Redundant sequences were eliminated and alignments were made using the ClustalW (Thompson *et al*., 1994). The evolutionary history of LotP was inferred by using the maximum‐likelihood method based on the JTT matrix‐based model. The phylogenetic tree with the highest log likelihood (−15397.4801) was constructed. The initial data for the heuristic search was obtained automatically by applying neighbor‐joining and BioNJ algorithms to a matrix of pairwise distances estimated using a JTT model. The topology with superior log‐likelihood value was selected. The branch lengths were measured based on the number of substitutions per site. The analysis involved 96 amino acid sequences. All positions containing gaps and missing data were eliminated. Evolutionary analyses were conducted in MEGA7 (Kumar *et al*., 2016). Further, the phylogenetic tree was constructed using the online tool Itol: Interactive Tree of Life (Letunic and Bork, 2016). The main LotP protein scaffold was modelled *in silico* using the automated mode of the SWISS‐MODEL and PHYRE2 server (Arnold *et al*., 2006; Kelley and Sternberg, 2009). The crystal structure of C3M65A from the N‐terminal domain of *Bacillus subtilis* Lon Protease was retrieved as the best template.

### Strains and growth conditions

Bacterial strains and plasmids are listed in Table 2. *E. coli* strains were grown in either LB medium or M9 minimal medium with 0.2% glycerol as the carbon source. *S. meliloti* cells were grown at 30°C in Luria–Bertani (LB). When required, the media were supplemented with ampicillin (100 μg ml^−1^) or kanamycin (20 μg ml^−1^) for *E. coli*.

*Liberibacter crescens* BT‐1 was cultured at 25°C with agitation (200 rpm) in modified BM7 media, pH 6.9 (Leonard *et al*., 2012). This culture medium was composed of 1% brain heart infusion (Difco Laboratories, Detroit, MI), 15% fetal bovine serum (Sigma‐Aldrich, St. Louis, MO), 30% TMN‐FH insect medium (Sigma), α‐ketoglutaric acid (2 mg ml^−1^), ACES (10 mg ml^−1^) and potassium hydroxide (3.75 mg ml^−1^).

### DNA manipulations and gene cloning

Standard methods were used for chromosomal DNA isolation, restriction enzyme digestion, agarose gel electrophoresis, ligation and transformation (Sambrook *et al*., 1989). Plasmids were isolated using QIAprep^®^ Spin Miniprep Kit (Qiagen, Valencia, CA, USA), and PCR products were purified using QIAquick^®^ purification kits (Qiagen). The primers utilized are described in Table 4. *Liberibacter asiaticus* DNA was isolated from leaf tissues of infected plants using the Isolate II Plant DNA kit (Bioline, Taunton, Massachusetts, USA).

**Table 4:** Oligonucleotides used in this study

Primers	Oligonucleotide sequence (5′→3′)
CLIB_03135_Ext_Fw	CAAGGTTGCGTCGTATCTTGA
CLIB_03135_Ext_Rv	CCCTGATCATCTACCTGACGC
CLIB_03135_Fw	CAAGCTTCGTCATCATTGCAACCTATTTTCACAAT
CLIB_03135_Rv	TTGTATTTCCAGGGC ATGAAAATTGGTAATACAATATACA
CLIB_03135_NdeI_Fw	GGATCCATATGATGAAAATTGGTAATACAATATACAAA
CLIB_03135_NotI_Rv	TATATGCGGCCGCTTGCAACCTATTTTCACAATGA
CLIB_03135_KpnI_Fw	CGATGTGGTACCTATGAAAATTGGTAATACAATA
CLIB_03135_SalI_Rv	TTCTACGTCGACTATTGCAACCTATTTTC
CLIB_03720_Ext_Fw	GGTGACATTGTCCTTTTTGG
CLIB_03720_Ext_Rv	AGCTTCCTTCTCCCATAAGC
CLIB_03720_NdeI_Fw	GGAATTCCATATGGGTGTTAATACGCTTGC
CLIB_03720_NotI_Rv	TATATGCGGCCGCCATCATCATATCCATACCAC
16SLcr_Rv	AAGGTTGAGCCTTGGGATTT
16SLcr_Fw	GTTCGGAATAACTGGGCGTA
CLIB_03135_EMSA_Fw1	GCAAGGTTGCGTCGTATCTT
CLIB_03135_EMSA_Rv_Bio^a^	CGGTAAGTCTTCACGATTTTTG

**a**. Biotin labelled.

### Protein purification

*CLIBASIA_03135* was cloned into the p15TV‐L vector and transformed into *E. coli* DH5α. The correct cloning was confirmed by sequencing. The recombinant plasmids selected were used to transform *E. coli* BL21‐Star (DE3) cells (Stratagene). The cells were grown in LB broth at 37°C. His_6x_‐tagged protein was induced with 0.5 mM IPTG when OD_600_ = 0.5. After addition of IPTG, cells were incubated at 17°C overnight with shaking. The cells were harvested and the pellet was suspended in binding buffer (500 mM NaCl, 5% glycerol, 50 mM Tris pH 8.0, 0.25 mM TCEP and 5 mM imidazole) and stored at −80°C. The cells were disrupted using a French Press, and the recombinant protein was purified as described in Pagliai *et al*. (2014). The purified proteins were dialysed against 50 mM Tris pH 8.0, 150 mM NaCl, 10 mM MgCl_2,_ 5% glycerol, 0.25 mM TCEP and stored in aliquots at −80°C until further use. Protein concentration was estimated using the Bio‐Rad protein assay kit (Bio‐Rad). LdtR was purified as previously described (Pagliai *et al*., 2014).

### Size‐exclusion chromatography

A solution containing 36–40 μM of recombinant LotP was prepared using 50 mM Tris pH 8.0, 150 mM NaCl, 10 mM MgCl_2_ buffer. Aliquots of this preparation were injected onto a prepacked Superose 12 10/300 chromatography column (GE) equilibrated with the same buffer. The gel filtration assay was carried out at 4°C using a flow rate of 0.3 ml min^−1^. The eluted proteins were monitored continuously at 280 nm using a UV‐M II monitor (GE Healthcare, United Kingdom). A mixture of protein molecular weight standards, containing thyroglobulin (670 kDa), γ‐globulin (158 kDa), ovalbumin (44 kDa), myoglobin (17 kDa) and vitamin B12 (1.36 kDa), was used in similar conditions as native molecular mass markers. The molecular weight of eluted proteins was determined using a calibration curve based on the retention time of each marker used.

### Immunoprecipitation

Protein–protein interaction assays were carried out using *L. crescens* or *E. coli* cell‐free extracts as indicated. His_6X_ antibody (Ab) was added to previously separated Protein G Dynabeads (Life Technologies (Acquired by Thermo Fisher), Waltham, Massachusetts, USA). After the Ab binds to the Dynabeads, the pearls with the Ab attached were collected using a magnet. The Ab was cross‐linked to the Dynabeads to avoid co‐elution of the antibody, and the magnetic beads were re‐purified. The beads were re‐suspended and incubated in a rotary shaker for 15 min in the presence of the recombinant protein (His‐LotP) and the bacterial lysate to favour the antigen (Ag)–antibody reaction. The final protein ratio used was 0.5 mg ml^−1^ His_6X_‐LotP and 750 μl of cell lysate with 1.5 mg ml^−1^ of total protein concentration. The mixture was washed in a buffer three times, separating the complex with the magnets between washes. The supernatant was removed and the Dynabead–Ab–Ag complex was transferred to a clean tube. The proteins were eluted from the magnetic beads and aliquots analysed in SDS‐PAGE. The protein bands were visualized with CBBR‐250, excised from the acrylamide gel and used in mass spectroscopy analysis.

### Mass spectroscopy

All MS/MS samples were analysed using Mascot (version 2.4.1; Matrix Science, London, UK). Mascot was set up to search the NCBInr_20130403 database (selected for Bacteria, unknown version, 14961948 entries) assuming the digestion enzyme trypsin. Mascot search was set with a fragment ion mass tolerance of 0.50 Da and a parent ion tolerance of 10.0 PPM. Scaffold (version Scaffold_4.4.8; Proteome Software Inc., Portland, OR, USA) was used to validate MS/MS‐based peptide and protein identifications. The identification of the proteins was accepted if at least two peptides match a score of 80.0% by the Peptide Prophet algorithm with Scaffold delta‐mass correction.

### Enzymatic activities

Phosphate released after enzymatic ATP hydrolysis was quantified using the malachite green assay in order to assess ATPase activity (Crowe *et al*., 2010). Protease activity was assessed by using azocasein as the substrate in the presence and absence of ATP and using proteinase K as a positive control (Secades and Guijarro, 1999).

### Growth curves

Growth kinetics using *E. coli* strains were carried out in M9 media with 0.2% glycerol as the sole carbon source. Each strain harbouring a plasmid was cultured in the presence of the corresponding antibiotic. Individual flasks were inoculated with aliquots obtained from an overnight culture with the necessary amount of cells to reach an initial OD_600_ = 0.05. The inducer (10 mM l‐arabinose) was added to the culture at the beginning of the assay. The flasks were incubated at 200 rpm at the indicated temperatures (37 or 42°C). In all cases, the growth curves were performed in triplicate.

### Two‐hybrid system

The protein–protein interaction between LotP and GroEL was assessed by cloning the genes encoding the interacting proteins in a two‐hybrid system (Vallet‐Gely *et al*., 2005). The assay was carried out using *E. coli* as previously described (Wrench *et al*., 2013). Briefly, *CLIBASIA_03135 and CLIBASIA_03720* genes from *L. asiaticus* were PCR‐amplified and fused to the ω subunit of the RNAP by cloning the fragment into the NdeI and NotI sites of the pBRGP‐ω plasmid. The *CLIBASIA_03135 and CLIBASIA_03720* genes were also fused to the zinc finger DNA‐binding protein of the murine Zif268 protein cloned in the same restriction sites of the plasmid pACTR‐AP‐Zif. The recombinant clones were selected by transformation in *E. coli* JM109, confirmed by sequencing and transformed into the reporter strain KDZif1ΔZ by standard methods (Sambrook *et al*., 1989). The recombinant *E. coli* cells were grown at different temperatures in MOPS media amended with 2 g l^−1^ glycerol as the main carbon source. The expression of the chimerical proteins in the reporter strain was induced by the addition of 20 μM IPTG at the beginning of the assay. Cell samples obtained at different culture times were permeabilized with 0.15% SDS and 1.5% chloroform in Z‐buffer (Miller, 1972). β‐Galactosidase activity was assayed using chlorophenol red‐β‐D galactopyranoside (CRPG). The substrate hydrolysis was continuously monitored, reading the absorbance at 570 nm for 10 min in a Synergy HT 96‐well plate reader (BioTek, Winooski, VT, USA). The β‐galactosidase activity, expressed as arbitrary units (AU), was calculated using the slope of the first‐order rate kinetic (V_0_) normalized to the cell density of each sample. The assays were performed in triplicate.

### Electrophoretic Mobility Shift Assays (EMSAs)

DNA gel shift assays were performed according to the protocol described in Pagliai *et al*. (2014). A fragment of the *CLIBASIA_03135* promoter was generated by PCR using biotin‐labelled primers (Table 4) and then purified using QIAquick spin columns (Qiagen). Briefly, the reaction for EMSA contained 1 ng of 5′‐labelled DNA probe, 50 mM Tris–HCl pH 7.2, 150 mM KCl, 10 mM MgCl_2_, 0.01% Triton X‐100, 12.5 ng μl^−1^ of an equimolar mix of Poly(dI‐dC) and Poly(dA‐dT) non‐specific DNA competitor and purified LdtR protein (0 – 500 nM). The whole reaction was incubated at 37°C for 20 min and then separated on 6% acrylamide–bisacrylamide non‐denaturing gels using 0.5× Tris–borate–EDTA buffer, pH 8.3 (TBE). Samples were subjected to electrophoresis for 2 h at 100 V using ice‐cold 0.5× TBE buffer, and then, the DNA was transferred from the polyacrylamide gel to a Hybond‐N^+^ membrane (GE Healthcare) by blotting at 250 mA for 45 min using a semidry transfer unit, followed by a UV cross‐link of the DNA to the membrane. The biotin‐labelled DNA was visualized using the Phototope‐Star detection kit (New England Biolabs, Ispwich, Massachusetts, USA).

### qRT‐PCR studies

*Liberibacter crescens* cells were grown at 200 rpm in BM7 media at 25, 30 and 32°C. The cells were harvested by centrifugation at 4°C at different points of the growth curve. Total RNA was isolated using the RiboPure‐Bacteria kit (Ambion, (Owned by Thermo Fisher), Waltham, Massachusetts, USA). The cDNAs were synthesized using the iScript cDNA Synthesis kit (Bio‐Rad, Hercules, CA, USA) using random hexamers, in accordance with the manufacturer's protocol. Quantitative real‐time PCR was performed in a Bio‐Rad iCycler IQ apparatus, using the iQ SYBR Green SuperMix (Bio‐Rad). The sequences of primers for B488_12950 and 16S RNA, used to normalize the expression values, are depicted in Table 4.

### Statistical analysis

Data analysis was performed using the statistical analysis software program R. A two‐tailed Student's *t*‐test was used to determine the statistical significance of the qRT‐PCR data. The fold changes in expression were normalized to the 16S rRNA control. The statistical significance of β‐galactosidase activities was determined with an analysis of variance (ANOVA) and Tukey's HSD *post hoc* test. ANOVA was completed to analyse the overall variation in mean activity. Tukey's HSD test was applied in order to specifically determine which strains exhibited the observed variation in mean activity. A *P*‐value < 0.05 was considered statistically significant for all analyses and α = 0.05 was implemented for Tukey's HSD testing. All assays were completed in a minimum of triplicate.

## Conflict of Interest

The authors declare that the research was conducted in the absence of any commercial or financial relationships that could be construed as a potential conflict of interest.

## Author contributions

FL, JFC and CFG designed, performed and analysed the experiments. KAP, FAP and CLG provided assistance and contributed in the preparation of the manuscript. JFC, FL and CFG wrote the paper. CFG and GLL conceived and coordinated the study. All authors reviewed the results and approved the final version of the manuscript.

## Supporting information


**Fig. S1**. Immunoprecipitation assays.

**Fig. S2**. Immunoprecipitation assays.

**Fig. S3.** Determination of LotP native molecular weight.
