# Supplementary figures and images for: Functional characterization of LotP from Liberibacter asiaticus
Source: Microb Biotechnol. 2017 Apr 5;10(3):642–56. doi: 10.1111/1751-7915.12706 (PMC5404198; doi:10.1111/1751-7915.12706)

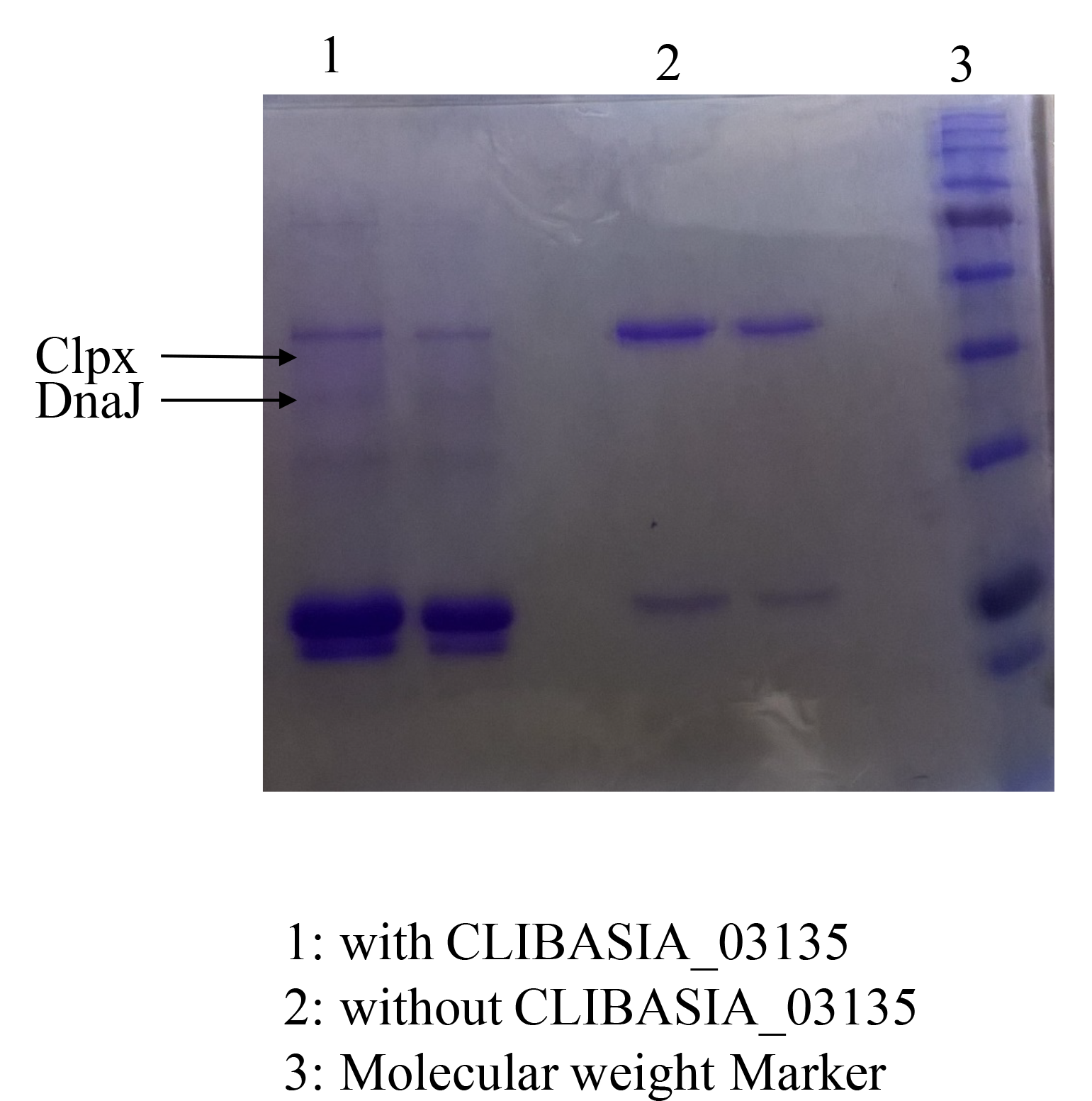

Supplement: Supplementary file 1 — Fig. S1. Immunoprecipitation assays. [file MBT2-10-642-s001.tif]

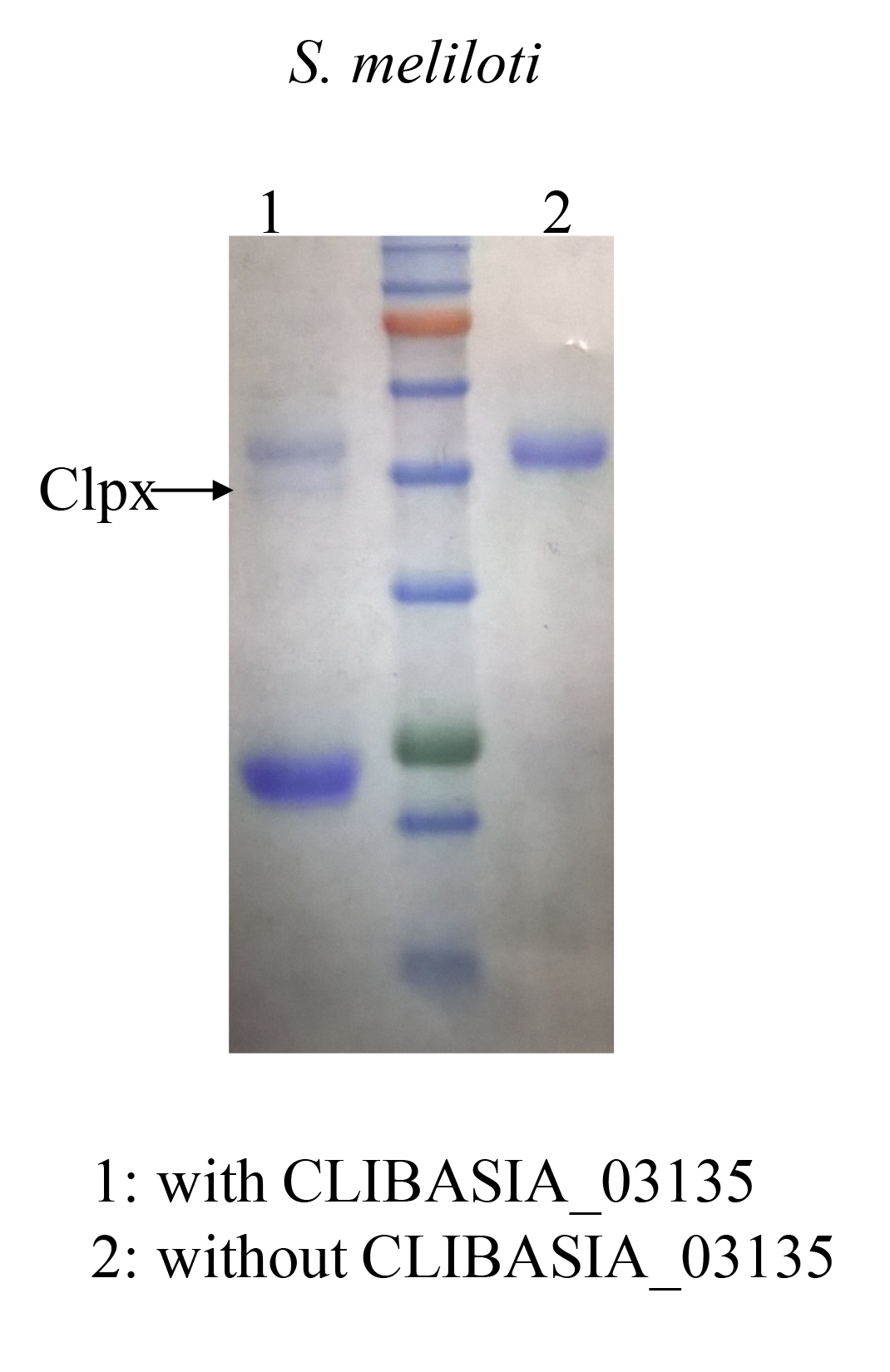

Supplement: Supplementary file 2 — Fig. S2. Immunoprecipitation assays. [file MBT2-10-642-s002.tif]

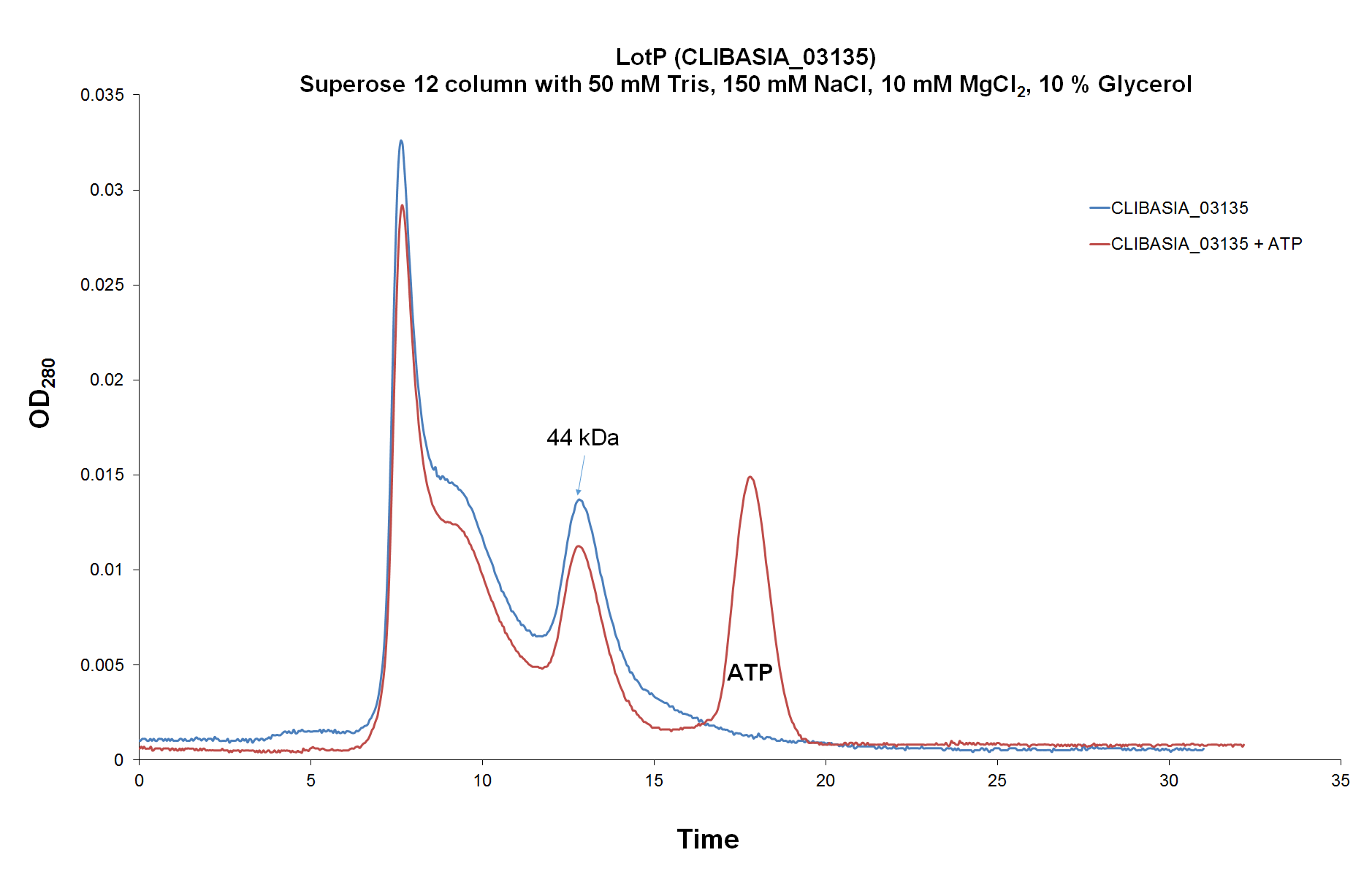

Supplement: Supplementary file 3 — Fig. S3. Determination of LotP native molecular weight. [file MBT2-10-642-s003.tif]
